# Hospital and Institutional News

**Published:** 1921-01-01

**Authors:** 


### HOSPITAL AND INSTITUTIONAL NEWS.

### CHRISTMAS IN THE HOSPITALS.

Elsewhere we give local descriptions of the individual Christmas festivities of some of the London and provincial hospitals (p. 311), and it is impossible, in a few words, to convey a picture of exactly what all these efforts to produce happiness for others imply. Above all things, the visitor could everywhere see an unrivalled demonstration of good fellowship and that true sympathy which the sick man deserves; all, in fact, that is best in human nature. No one can pick out a single hospital staff for especial praise. Local conditions and opportunities are so varied that the onlooker must accept our word that in every instance the maximum has been accomplished, and that with the utmost self-sacrifice on the part of the workers concerned. The arrangements made for London were on a decidedly more elaborate scale than in recent years, and for that we have chiefly to thank the passing of war and, in the hospitals, part at least of its aftermath. In the typical instance every ward was profusely but artistically decorated with holly, evergreen, and flowers received from the hospital's numberless friends. Sisters, nurses, the resident staffs, and the patients have, willingly supported by outside resources, worked with almost unprecedented zeal to bring the true Christmas atmosphere to every corner of their institutions. Literally almost, every child in hospital this Christmas has received a present from a Christmas-tree. Every possible ward was supplied with a piano. Concert parties without number have gladdened the hearts of the patients, and minor festivities have made a series of Christmas hours pass with a swiftness which had to be experienced to be believed. One of the most beautiful customs is represented by the various types of out-patient receptions which the London hospitals provide for poor children. Last year we described at length the wonderful Landon teas at Guy's. At St. Bartholomew's eighty of the poorest children attending the hospital as out-patients are invited by the sister in charge of the surgery for Christmas morning when Father Christmas presents each with a parcel of clothes, a toy, and some fruit. But these simple instances cannot convey to the uninitiated one tithe of the wonders and beauties of the hospitals' Christmas. They must be seen to be understood, and once seen they will never be forgotten. To many of our readers a Christmas in such surroundings is, indeed, no new experience, but it is to those others that we appeal. At Christmas the hospitals' doors are open wide. The outside world is invited to view and to learn. We trust that in the days just past there are many who have power to help to whom an awakening has come. If any who read this note have still to experience Christmas as the hospitals make it, may we suggest that next year they pay the necessary visit? They will indeed be assured of a welcome.

### A THEATRE'S CHRISTMAS SUGGESTION.

The children patients in a number of the large London hospitals will benefit during the Christmas season by the suggestion made by the management of the Garrick Theatre that all children attending the performance of "The Shepherdess without a Heart" should bring with them a gift, to be placed on a large Christmas-tree in the lobby, and afterwards sent to one or other of their less fortunate brothers and sisters in hospital at this time.

### THE PASSING OF LOCAL INSURANCE COMMITTEES.

At the end of the year the local insurance committees for tuberculosis work under the National Health Insurance scheme ceased to function. In the large sanitary areas, at any rate, arrangements have been made for central insurance committees to take their place until May next. It is rumoured that this temporary plan has been decided on because the local sanitary authorities are not yet ready for the duty assigned, which, of course, will eventually fall to their lot. When that state of affairs has been realised a simplification of machinery and unification of control will have been reached such as has long been desirable. In particular the clerical work of tuberculosis officers will be somewhat lessened, with consequent increased freedom for clinical work. It is to be hoped that "after-care," long neglected by the local insurance committees, will in the future receive more attention, for such is urgently necessary. In short, decentralisation in tuberculosis administration has not proved a success.

### WILLIAM MARSDEN: HOSPITAL FOUNDER.

At a dinner recently given by the Medical Committee of the Cancer Hospital to some of their lay colleagues and other guests, the Chairman, Mr. Charles Ryall, F.R.C.S., recalled some of the details of the career of William Marsden. While a student at St. Bartholomew's, some eighty or ninety years ago, Marsden found a girl on the steps of a City church (St. Andrew's) dying of want and disease. He put her in a cab and drove her to St. Bartholomew's, but she was refused admittance, on the ground, apparently, that she had no subscriber's letter. Marsden then took her to two or three more hospitals, where also admittance was refused. He then found a lodging for her and had her cared for until her death, which shortly occurred. Shocked by the experience, he decided to found a hospital where no subscribers' letters should be issued; and he called this hospital the Free Hospital, a name afterwards changed to its present form—The Royal Free Hospital. Later in life his wife died of internal cancer, and this moved him to found a new hospital for the study of this disease, the present Cancer Hospital in the Fulham Road. Curiously enough, the efflux of time has seen his two hospitals forge a certain link of union; for an arrangement now exists by which students from the Royal Free Hospital can do part of their work in clinical surgery in the wards of the Cancer Hospital.

### THE PEOPLE'S LEAGUE OF HEALTH.

As a result of a meeting held by the People's League of Health at the Cardiff City Hall on December 15, under the Chairmanship of the Lord Mayor, at which Sir Bruce-Bruce-Porter, Dr. Eric Pritchard, Dr. Mary Scharlieb, and Dr. E. H. Stancomb, representing its Medical Council, were the speakers, a Committee was formed to establish the *work of the League in Wales*. Among those organising the Welsh Branch are Prof. Edgar Collis, Dr. Ewen J. Maclean, and Mr. D. W. Evans. The Committee, while expressing their very high appreciation of the aims and objects of the League, consider the present moment to be singularly opportune for the furtherance of the campaign undertaken by the League, particularly in the direction of developing a strong public demand in favour of health legislation.

### "THE LARGEST CHARITABLE FUND."

Engraved upon a silver salver presented to Sir Robert Hudson by his colleagues on the Joint War Finance Committee of the British Red Cross Society and the Order of St. John was engraved the following inscription: "Presented to Sir Robert Arundell Hudson, G.B.E., by the members and secretary of the Joint War Finance Committee, British Red Cross Society and Order of St. John, 1914-1920, in grateful remembrance of their chairman's impartiality and unwearied patience, as well as distinguished ability and judgment, in the transaction of business involving an expenditure of 20 millions sterling on behalf of the sick and wounded sufferers in the great war." In making the presentation, Sir William Goschen referred to the skill with which Sir Robert had handled the largest charitable fund ever raised in this country.

### SURPLUS ASSETS FOR DISTRIBUTION.

According to an announcement in the *London Gazette* of December 28, any institutions claiming to have objects similar to those of the British Ambulance Committee, Service de Santé Militaire (which is in voluntary liquidation), are to come in and prove their claims, on or before January 31, at the Chambers of the Registrar, Companies (Winding-up), Bankruptcy Buildings, Carey Street, London, W.C. The hearing and adjudicating upon the claims, for the purpose of ascertaining whether there are any institutions entitled to participate in the surplus assets of the British Ambulance Committee, is fixed for Monday, February 7. The objects of the Society are set out in the *London Gazette* of December 28, and the Liquidator is Francis W. Pixley, c/o Burchells, 5 The Sanctuary, Westminster, S.W.

### FIRST FRUITS OF "OUR DAY."

Four thousand pounds to the League of Mercy and £7,500 to the Hospital Saturday Fund have been forwarded by the Joint Council of the Red Cross Society and Order of St. John in accordance with an agreement made in respect of the co-operation of these organisations on "Our Day," the sums mentioned being for distribution amongst the hospitals. The statement of the result of the great Red Cross effort on October 14 last is awaited with much interest.

### THE WORK OF DENTAL HOSPITALS.

Whether the reason be the growth of medical treatment of school children, or whether all classes are less indifferent to dental caries than they used to be, the work of most dental hospitals is expanding quickly. In the North of Scotland, for instance, the number of visits paid by patients to the Dundee Dental Hospital has risen to over 3,000 in the past year, and the Chairman, Mr. William Boyd, confidently predicts that by the end of 1921 the number of attendances will have risen to 4,000. Unfortunately, the past year showed also a small deficit, and Mr. D. Robertson, the secretary, and the Finance Committee are busily engaged by the need for increasing the income. While the patients grow in numbers, the students at the hospital also are increasing, and every hope in this quarter at least has been fulfilled. Scotland has certainly gained by the training given to students at the Dundee School, which is, of course, connected with the diploma now obtainable at St. Andrew's.

### LAY NEWSPAPERS AND MEDICAL NEWS.

We all have seen in the daily Press from time to time accounts of marvellous cures and discoveries, and have noticed that they are seldom heard of again. The latest was an American cure for epilepsy by operation, the account of which gave considerable amusement to medical circles. Dr. Georges Bourgeau, in a recent work, complains of sensational headlines in the French lay Press, and quotes the following: "Contagion Has Been Abolished in Scarlet Fever"; "The Transfusion of Blood is a Dangerous Procedure for the Donor"; "Arteriosclerosis is Vanquished"; "One Half of Those who are Deaf are Curable." "Why does the journalist commit so many mistakes in matters medical?" asks Dr. Bourgeau. Perhaps it is because he sees in a discovery an article to be written, a chance for a sensational heading, and, in the haste to be the first to announce it, abandons all effort for accuracy as to either facts or time.

### ST. MARY'S WEEK.

It is a new and pleasing sign that medical students are interesting themselves in the income of the hospitals to which they are attached. Following, no doubt, the example of the London Hospital medical students, the students' section of St. Mary's Hospital Appeal Fund arranged a St. Mary's Week. Recently they proposed to visit the chief restaurants, hotels, theatres, shops, music-halls, cinemas, and even railway stations, where, with official sanction, they collect whatever they could. The proceeds will be divided between the general purposes for which the appeal was devised and the endowment of the new Chair of Industrial Medicine attached to the hospital. We hope that the result will have justified the enthusiasm of the band of collectors.

### GUY'S AND CITY FIRMS.

A reasonable measure of success has attended the preliminary effort of Guy's Hospital to collect regular contributions from the firms in its immediate neighbourhood. In the past few months nearly £9,000 has been raised from these firms, whose contributions are reckoned by the authorities as a business expense. We trust that the above sum, which compares well with the meagre £3,500 supplied by annual subscriptions, will not only prove an annual source of income, but a growing permanent receipt. The Prince of Wales has given his support to the scheme, and heads the list with a personal subscription.

### TRADESMEN'S FUNDS IN LONDON.

We are glad to see that the success of the Deptford Tradesmen's Fund, which has raised £534 for the benefit of the Miller Hospital, Greenwich, has led to the formation of a Hospital Aid Society, which came into being when the tradesmen's fund was closed. At a meeting when ten life governorships were conferred upon the active workers of the Deptford Tradesmen's Fund, several tradesmen from Greenwich were present, and there seems some chance that a similar tradesmen's fund may now spring up in Greenwich also. All this is part of the monument for special funds for every class, and for the reinforcement of weekly contributions from the "workers" by a similar organisation for other bodies besides workmen. There is more hope in this movement than in special appeals, which should only be used for special needs, and not, as hitherto, for the principal source of income. We hope that the example of Deptford and Greenwich will be followed in other London boroughs. Every instance is worth recording, because each helps to show that all districts can adopt the plan if the attempt is made in earnest.

### A MUNICIPAL CONVALESCENT HOME.

One of the first of the convalescent homes under the 1918 Maternity Act has been opened by the Hertfordshire County Council at St. Leonards-on-Sea, and already the admissions have been seven convalescent mothers with their babies and twenty-two young children. Each patient is admitted, in the first instance, for a period of three weeks, but remains longer if so recommended by the visiting physician. A clear set of rules has been drawn up for reasons of discipline, but as these are of the simplest character they are not irksome. The result is that the children and mothers appear to be very happy in the home, while the excellent effect of the sea air, proper and regular feeding, rest from work and care, and other benefits derived from a stay in the home, is apparent. The County Council reports that the home will prove of great value in restoring the health of delicate children and of delicate mothers after confinement.

### THE AEROPLANE AND HEALTH.

The discovery has been made that the aeroplane can be utilised by municipal authorities for purposes of health. It is, of course, valuable for town-planning and development, for which purposes an aerial survey is invaluable. Calcutta Corporation has realised that an aerial survey would assist the health department, especially in enabling the location of areas liable to be waterlogged—especially if the survey were made during the rainy season. The Council is accordingly ascertaining the cost of such an aerial survey.

### WALLASEY COTTAGE HOSPITAL.

The Bishop of Chester has opened the new wing added to Wallasey Cottage Hospital by the generosity of Mr. Herbert E. Wild, chairman of the hospital committee, in memory of the men of the neighbourhood who gave their lives in the war. The building will be used chiefly to increase the accommodation of the nurses, who are provided with five extra bedrooms, dining-room and a drawing-room. There are also four private wards. Dr. Bouverie McDonald, M.P., said that the institution, whose progress he has watched for thirty-four years, will soon have outgrown the description of a cottage hospital.

### THE HELPER HELPED.

"We need your help, you may need ours" was a popular slogan at one time much in use by a certain ambulance organisation, and how true this is in regard to hospital work was recently shown in an unfortunate accident which befell Captain Brockwell, who sustained serious injury when making preparations for an entertainment to be given in aid of the Willesden Hospital, and had to be removed to the very institution he was endeavouring to assist.

### TUBERCULOSIS RESEARCH.

Islington Borough Council is asking the Ministry of Health to consider the advisability of instituting a Department of Research specially to find the means of effectually preventing the spread and development of tuberculosis. The Council decided to do this as a consequence of the report from its delegates at the recent conference on tuberculosis, when there was a consensus of opinion that sufficient means were not available for scientific study and research.

### HOSPITAL SUPPORT IN MANCHESTER.

Speaking at the annual meeting of the Manchester and Salford Charities Fund, Sir Henry Miers, the Vice-Chancellor of Manchester University, said that an extraordinarily critical time for hospitals had been reached. It is therefore gratifying to see an increase in the collections. That for the Sunday Fund is £1,400 more than in 1919. The city and borough districts contributed £500 more than hitherto, but their total is still less than the cost of treating the patients from their districts. The fund, however, has begun a new phase of usefulness by the gift of £25,000 from the late Mr. Jesse Haworth. This, which has been invested in War Stock, will produce an income of £1,250. The money is in the hands of the Official Trustees for Charitable Bequests.

### NOVEL MEANS OF IDENTIFICATION.

Some short while since we noted the taking of footprints as the means of identifying children abroad; another unique method seems now to have been adopted in America, where for the purpose of identification deaf and dumb children in some of the institutions are stated to have their names inscribed on their necks—presumably in a position out of sight!

### PAYING PATIENTS AND PROVIDENT PLANS.

Those who have followed the history—the financial history in particular—of the Royal Sussex County Hospital during the past decade can readily understand the unanimous verdict which the governors have just given for the adoption of a paying patients' scheme. Despite the greatest economy in every direction year by year, the governors have been faced with a deficit which has required immense personal efforts to liquidate. Even threats to close down some of the wards have not brought in the subscriptions from those who really ought to give, and give freely. And so the governors have unhesitatingly followed the example of some other hospitals in the country in deciding to adopt a paying patients' system, in the hope that by so doing they can still carry on the largest hospital in the county of Sussex as it should be, and uphold the great reputation it has established for itself. At the governors' meeting, when the necessary sanction was given to the new statute, no mention was made of the charges which will be made, but as a corollary to the scheme the "blessing" of the governors was given to a local provident scheme for hospital benefits and additional medical services. This is quite apart from the benefits which can be derived under the National Health Insurance. The first 10,000 members in this provident scheme will receive an original member's card, and their subscription will not be raised. How these cards are taken up will reflect the view of Sussex people in the scheme, and not only in the hospital world, but in a far wider field, this scheme will be watched with considerable interest.

### A PAY HOSPITAL FOR BRISTOL?

A movement has begun in favour of providing a hospital for paying patients at Bristol. The members of the medical staffs of the Royal Infirmary, the Royal Hospital for Children, and the Eye Hospital lately passed a resolution stating that "a completely equipped hospital is urgently needed in Bristol for all classes of patients who are able to pay for medical and surgical attendance in addition to charges for maintenance." The *Bristol Medico-Chirurgical Journal* has lent its support to the scheme, but at the time of writing the plan has not gone beyond discussion.

### A QUESTION OF SERVICE.

An unusual state of affairs is reported from Aberystwyth. The Board of Guardians, which is very largely composed of farmers, recently advertised for a medical officer for the borough portion of the Union, and among the applicants was Dr. Thomas, who served throughout the war with the Cardiganshire Battery. The Board, however, did not appoint the ex-Service candidate, which aroused some feeling locally, culminating in public meeting protests. These eventually went to the Ministry of Health, which thereupon took a hand in the matter. As the Guardians refused the Ministry's request that the appointment be reconsidered, Dr. Addison's Department has now instructed the Guardians' clerk that he must not pay any salary to the medical officer appointed by the Board.

### A HOSPITAL FOR CHEPSTOW.

Chepstow, which has gone ahead industrially very much during the past four or five years by reason of the shipyard established there, is to have a general hospital of its own. With commendable enterprise those at the head of the movement have secured for the very moderate sum of £2,550 Gwy House, a commodious residence which can be well and easily adapted to the needs of a small general hospital. Towards the purchase price the Monmouthshire Shipbuilding Company has subscribed £1,000, there is a surplus of £600 from the Chepstow Red Cross Fund, which is to go into the account, and a handsome donation is anticipated from the workmen's fund. A good start has therefore been made, and at a meeting last week the public gave its approval to the rules and regulations which have been framed for the government of the institution.

### A HITCH AT STOKE.

The Buildings Committee of the Stoke Board of Guardians has, in effect, reported against the plan to use the Guardians' institution to relieve the long waiting-list at the North Staffordshire Infirmary. The committee declares that when the proposed block shall have been altered, only 180 patients can be accommodated, and that the effect would be that 110 aged patients would have to be displaced and boarded out if there were to be any margin in reserve. This has led the hospital sub-committee of the Guardians to postpone the matter, and the North Staffordshire Infirmary has been informed of the result. In spite of the difficulties the Guardians on the whole favour co-operation, and it is still possible that something may be done.

### THIS WEEK'S DRUG MARKET.

The market has only just reopened, and business is in a stagnant condition, and likely to remain so until stocktaking is over. In some quarters a revival of activity seems to be expected early in the New Year, but it is really very difficult to see what is going to turn the tide. So long as prices continue to move downwards buyers will make purchases very sparingly, and at the moment it does not look as if the downward movement has received a check. In certain drugs for which Germany had a high reputation before the war competition is likely to go on, and that being the case, prices may fall still further. Values at the moment are more or less normal in the absence of business.

